# Posterior nasal packing: illustration by a simple model

**DOI:** 10.1308/rcsann.2014.96.1.76

**Published:** 2014-01

**Authors:** P Kulloo, R Lakhani

**Affiliations:** ^1^Heatherwood and Wexham Park Hospitals NHS Foundation Trust,UK; ^2^Peterborough and Stamford Hospitals NHS Foundation Trust,UK

**Figure 1:**
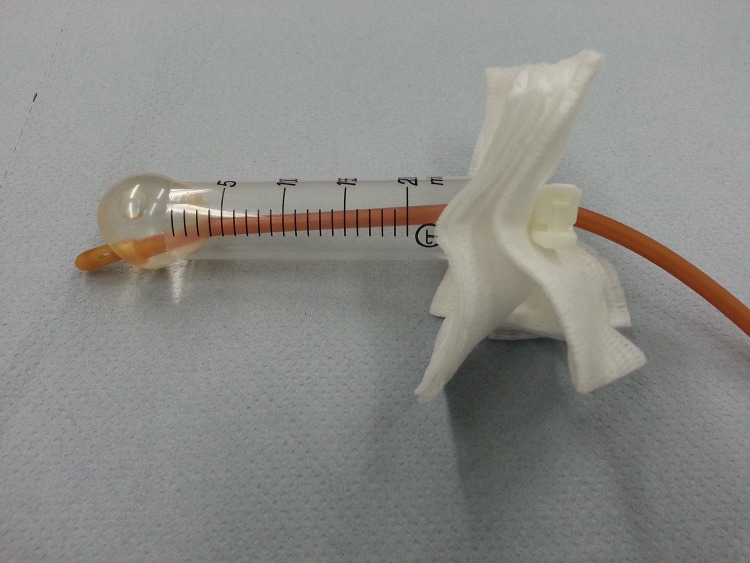
Posterior nasal pack

We designed a simple model to illustrate posterior nasal packing to inexperienced trainees in a classroom environment. The plunger and the smaller end of a 20ml syringe, which is cut off, are discarded. The hollow syringe represents the nasal cavity. A Foley catheter is inserted from one end until it exits at the other end, representing the catheter’s tip seen in the oropharynx. While pulling the catheter, the balloon is inflated until it rests against the syringe, corresponding to the posterior choana. The anterior end of the catheter is then secured with an umbilical clamp (Fig 1).

